# A SARS-CoV-2 minimum data standard to support national serology reporting

**DOI:** 10.1177/00045632241261274

**Published:** 2024-06-17

**Authors:** Esmond Urwin, Joanne Martin, Neil Sebire, Andy Harris, Jenny Johnson, Erum Masood, Gordon Milligan, Lucy Mairs, Antony Chuter, Michael Ferguson, Philip Quinlan, Emily Jefferson

**Affiliations:** 1 Digital Research Service, University of Nottingham, Nottingham, UK; 2 Centre for Genomics and Child Health, 4617 Queen Mary University of London, London, UK; 3 Institute of Child Health Population Policy and Practice, 573582 UCL Great Ormond Street Institute of Child Health, London, UK; 4 X-Lab Ltd., Leeds, UK; 5 School of Medicine, University of Dundee, Dundee, UK; 6 Public and Patient Involvement Group, 6123 University of Nottingham, Nottingham, UK; 7 School of Medicine, University of Nottingham, Nottingham, UK

**Keywords:** SARS-CoV-2, COVID-19, interoperability, data standards, laboratory data, serology, healthcare terminology, healthcare vocabulary

## Abstract

**Background:**

Healthcare laboratory systems produce and capture a vast array of information, yet do not always report all of this to the national infrastructure within the United Kingdom. The global COVID-19 pandemic brought about a much greater need for detailed healthcare data, one such instance being laboratory testing data. The reporting of qualitative laboratory test results (e.g. positive, negative or indeterminate) provides a basic understanding of levels of seropositivity. However, to better understand and interpret seropositivity, how it is determined and other factors that affect its calculation (i.e. levels of antibodies), quantitative laboratory test data are needed.

**Method:**

36 data attributes were collected from 3 NHS laboratories and 29 CO-CONNECT project partner organisations. These were assessed against the need for a minimum dataset to determine data attribute importance. An NHS laboratory feasibility study was undertaken to assess the minimum data standard, together with a literature review of national and international data standards and healthcare reports.

**Results:**

A COVID serology minimum data standard (CSMDS) comprising 12 data attributes was created and verified by 3 NHS laboratories to allow national granular reporting of COVID serology results. To support this, a standardised set of vocabulary terms was developed to represent laboratory analyser systems and laboratory information management systems.

**Conclusions:**

This paper puts forward a minimum viable standard for COVID-19 serology data attributes to enhance its granularity and augment the national reporting of COVID-19 serology laboratory results, with implications for future pandemics.

## Introduction

With the emergence of the coronavirus severe acute respiratory syndrome-coronavirus-2 (SARS-CoV-2) in late 2019 and the subsequent pandemic, the need to understand and study the virus became paramount.^1–4^ The ability to diagnose and understand a health threat and develop processes, behaviours, vaccines and therapeutics to fight it comes in part from the ability to amass, assess and analyse timely and meaningful data as quickly as possible.^5^ At the start of the pandemic, there was no comprehension of whether people who had contracted COVID-19 would be immune to later infection and, if so, how long immunity would last. Understanding serology and seroprevalence was key to determining how effective vaccines can be for both current and future variants.^6,7^ Vendors were developing new assays but could not calibrate the results as there was insufficient data on how to equate antibody binding units with immunity. Laboratories that were developing new assay techniques were not able to share or compare data between the differing techniques, compounding the problem.^8–11^

Within England, National Health Service (NHS) laboratories can send data by way of Labgnostic (The National Pathology Exchange, NPEx). The data that is sent varies lab to lab and health board to health board. There is no standard approach, nor is there a core minimum defined set of data attributes that are reported. This is in part due to the multitude of systems that exist for the creation and reporting of the data. There is no onward automated feed from Labgnostic to National Health Service (NHS) Digital (now NHS England), meaning that linkage to other relevant nationally collected health datasets does not take place routinely.

When the pandemic started, SARS-CoV-2 serology data was added to the data feeds sent from Test and Trace laboratories via Labgnostic to NHS Digital. However, data from NHS laboratories were not sent. High-level qualitative results were reported, that is, SARS-CoV-2 test results stating outcomes, those being either positive, negative or indeterminate. Although many laboratories produced and recorded quantitative data and results, very few reported this granular level data to Labgnostic, moreover, to be able to report this would have necessitated the development of new data pipelines to transfer the augmented SARS-CoV-2 test results to organisations such as NHS Digital.

The consistent reporting of qualitative data has proven a useful approach to understanding levels of seropositivity with the population; however, it is binary in nature (positive or negative) and reactive. To gain a greater understanding of the range of antibody levels within a seropositive population, and potentially gauge the consequences with respect to new SARS-CoV-2 variants, there was a need for more granular data to be reported from laboratories nationally.^12–14^ A good example of the minimum data variables (attributes) was described by the Centre for Controlled Disease,^15^ which stated 20 data variables as a core minimum to the recording and reporting of SARS-CoV-2 testing, together with associated guidelines.^16^ Additionally, the World Health Organisation (WHO) has put forward an International Standard (IS) for SARS-CoV-2, for which it states seven data variables assessed across a range of different SARS-CoV-2 S protein assays.^17^ They identified the limitation of sample sizes and recognised that more data was needed to further develop the standardisation approach and harmonisation efforts. The application of the WHO standard has shown that it promotes the ability to better analyse and compare immunology data across different datasets.^18^ However, emphasis was placed upon the need for ‘a standardised quantification of anti-SARS-CoV-2 antibodies’, being of ‘the utmost importance’.^18^ A call for action by the scientific community during 2022 called for more immunology data to help facilitate the comparison of quantitative assays results data to help better understand immune responses.^19^ Additonally, others also recognised this needed to be done to further develop SARS-CoV-2 data representation and standardisation to enable comparability across different testing methods and for population based studies too.^20–24^

The precise, unambiguous and standardised representation of data, its quality, subsequent harmonisation and interoperability is important as it constitutes the foundation for the accurate and meaningful analysis of multiple sources of data from different domains, institutions and countries.^25,26^ However, being able to collate, harmonise and represent data from different types of laboratory systems can be problematic.^27–33^ There are multiple variations that apply to laboratory testing procedures. Firstly, there are different assays that can be used to test for SARS-CoV-2 which, dependent upon the test being performed, produce different numerical outcomes.^3,34^ Such results are not easily comparable due to the difference in ranges and values between differing assay types, due to different target antigens and different detection technologies.^35–39^ Secondly, test kit batch variability within a given test type can introduce variance into the measurement process within laboratories.^40–42^ Thirdly, laboratory analyser systems themselves are subject to variance, this can in part be due to how machines are setup and calibrated thus producing bias and could include analytical imprecision.^43,44^ Lastly, there are a variety of different Laboratory Information Management Systems (LIMS), which record and represent data generated from the laboratory analyser systems. Moreover, these can be individually configured for the purpose at hand; thus, variation can exist between different instances of the same LIMS software, that is, laboratory A’s LIMS software configuration could be different from that of laboratory B, even though they both use the same piece of LIMS software.

When studied from a holistic viewpoint, this equates to multiple sources of variance throughout the laboratory testing process.^43,45^ To be able to fully understand and grasp how such variance exists and occurs throughout the testing process, the data must be accurate in the first place; moreover, the representation of the data itself must be formal and standardised.^46–49^ It is crucial that the description of each data attribute is unambiguous and explicit, so that it precisely describes what it represents, thereby removing the ability to misinterpret its meaning.^50–52^ Thus, the use and application of naming and naming conventions are paramount to present a standardised approach and, where possible, foster and enable interoperability.

Many laboratories name data attributes according to their own specifications and needs, thus producing local encoded messages. Whilst this is perfectly fine within the context of a singular or group of laboratories that utilise this code, when trying to share or interpret such encoding outside of that localised context, for example, nationally, it can be incredibly difficult to decipher such local naming conventions. Thus, trying to glean what testing kits and platforms are being used and how the actual results have been arrived at from reported laboratory data presents a serious problem.

Definitions and standards for data attributes and specification for many laboratory assays and test results are available within the United Kingdom, including specifications for interoperability via Labgnostic (e.g. Health Level 7 messaging).^53–56^ Additionally, there are standardised naming conventions for assays and test kits. However, the thorough and correct application and usage of such standards varies between laboratories.

The authors of the paper were involved in a range of initiatives across the country responding to the pandemic for example the National Core Studies Programme and found that granular COVID-19 data was not captured nor was it available for the Scientific Advisory Group for Emergencies (SAGE) to help answer pandemic questions.^4^ Sir Michael Fergusson chair of UK Health Security Agency (UKHSA) Scientific Advisory Group for antibody testing which, in its final lessons-learned recommended that pandemic preparedness should include ‘The ability to safely and quickly link serological/immune surveillance data to clinical and genomic data to answer research questions that can inform public health policy’.^57^

The CO-CONNECT project was a 2-year research programme funded by the Medical Research Council and the Department of Health and Social Care to build a federated platform streamlining the process for researchers to find and access COVID-19 related datasets from around the United Kingdom.^58,59^ Serology data was key to informing the COVID-19 response, with researchers requiring access to granular level serology data linked to relevant longitudinal healthcare records. This paper presents a defined minimum set of standardised data attributes for the reporting of SARS-CoV-2 laboratory results developed by the CO-CONNECT project, together with a set of formalised names for laboratory analyser systems, LIMS software providers and SARS-CoV-2 serology measurements as part of the CO-CONNECT standardised vocabulary.

## Method

**Figure 1.**
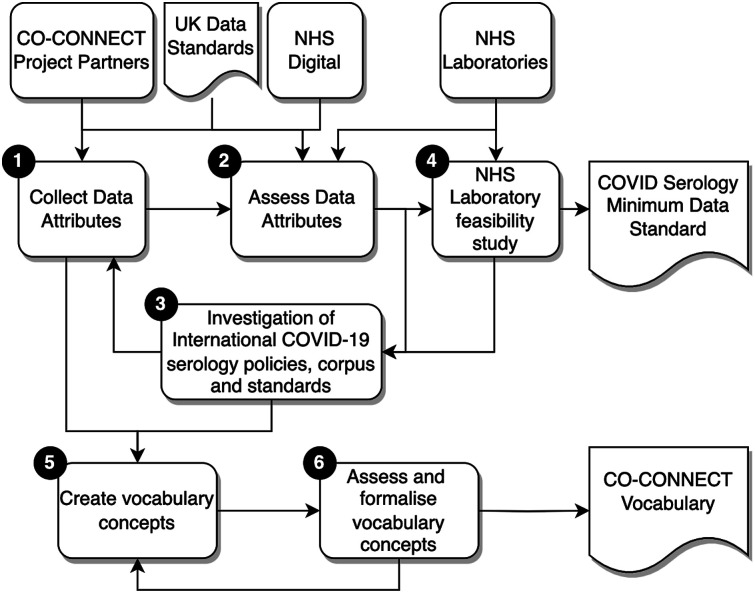
Co-Connect COVID-19 serology data standard development methodology.

The method applied for the COVID-19 serology minimum data standard (CSMDS) is illustrated in Figure 1. An inductive mixed methods approach was adopted utilising Delphi and teach-back qualitative methods.^60–64^

**Figure 2.**
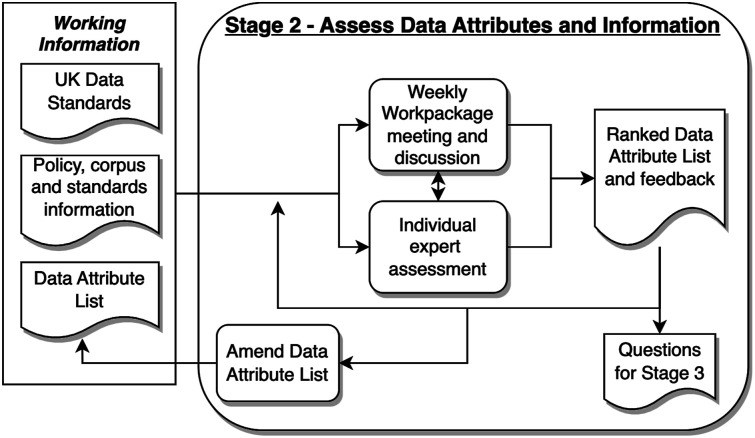
Stage 2 - assess data attributes and information processes.

The first three stages formed an iterative feedback process loop, to enable the development of a representative set of data attributes for a minimum data standard. The fourth stage was a laboratory feasibility study to assess the CSMDS. Stages 5 and 6 were used to develop the CO-CONNECT vocabulary. The six stages employed in the method were thus:

• Stage 1: Collection of data attributes: The data came from the CO-CONNECT partners organisations and three NHS laboratories. Each of the CO-CONNECT partners were collecting relevant COVID-19 serological data, from research cohorts and unconsented longitudinal datasets. After the relevant access permissions were gained from each organisation, metadata was extracted from anonymised data exports utilising the ODSHI White Rabbit statistical profiling tool^65^ and shared with the CO-CONNECT team for analysis. Three NHS laboratories at Barts Health NHS Trust (Barts), NHS Great Ormond Street Hospital for Children (GOSH) and NHS Tayside acted as exemplar sources of laboratory data, providing excerpts of data being reported. These different data feeds provided an initial set of 36 individual data attributes.
• Stage 2: Assessment of data attributes. Figure 2 depicts the approach taken for Stage 2, to which a Delphi method was used to help reach a consensus.^61,66,67^ Weekly workpackage meetings enabled debate and feedback against progress utilising expert knowledge from the project group, together with findings from stage three and UK data standards. Individual expert assessments took place independently of weekly meetings. From these processes ranked data attribute lists were produced together with collected expert feedback. The teach-back method was used, combining views and Delphi ranking votes, to reach a consensus. The iterative approach enabled the group to focus on the importance of data attributes within the standard highlighting those that were considered critical for reporting granular data. Those that were not were removed from the standard. Questions were collected to be answered in Stage 3.
• Stage 3: Investigation of International COVID-19 serology policies, corpus and standards: To further support the development of the CSMDS, other sources of information were studied, such as national standards, international standards, work from other organisations, other nations and the corpus of literature upon the subject.^15,16,49,54,55,68–70^ These fed into Stages 1 and 2 to inform the debate and further refine the approach.
• Stage 4: NHS laboratory feasibility study: Once the CSMDS had reached a level of maturity, the next stage was to understand whether it represented the COVID-19 serology test result data being collected within the CO-CONNECT partner organisations and pilot NHS laboratories and if it was fit for purpose. To accomplish this, a 4-step process was applied to each of the pilot laboratories at NHS Barts, NHS GOSH:
  Step 1: Assessment of laboratory HL7 messages – Anonymised excerpts of HL7 COVID-19 serology messages from the three laboratories were studied to understand how they represented the test data within the HL7 message syntax.
  Step 2: Comparison of laboratory HL7 messages against the CSMDS - the CSMDS was compared against each laboratory HL7 COVID-19 serology message to determine which CSMDS data attributes were represented and which were not, in effect a gap analysis was performed. The missing data attributes were highlighted.
  Step 3: Definition of HL7 changes – Utilising the highlighted missing CSMDS data attributes, changes were defined for each of the laboratories' HL7 messages so that they could be represented within them. In effect, where to put each data attribute within the message and accordingly the data type to use.
  Step 4: Laboratory assessment of the proposed HL7 changes – each of the three NHS laboratories assessed the proposed changes to their existing HL7 COVID-19 serology messages to move towards CSMDS standardisation. Specifically: (a) was it possible to make the necessary changes to their LIMS software and output the data attributes needed for the CSMDS; and (b) how easy was it to accomplish these changes and were there any complications that might hinder the move towards CSMDS standardisation?
• Stage 5: Creation of vocabulary concepts: Data attributes and exemplar HL7 laboratory messages were collected throughout the process of developing the CSMDS, these were examined to understand the types of laboratory analyser systems and LIMS software that were being used to capture the data. To support this, a web-based evaluation of current analysers and LIMS software was undertaken, together with a study of numerous hospital trust ISO 15189 accreditation certificates.^71^ From these, a list of laboratory analyser systems and LIMS software was compiled with the associated manufacturers.
• Stage 6: Assessment and formalisation of vocabulary concepts: The CO-CONNECT project data team reviewed the entries within the list of analysers and LIMS software and assessed whether they were suitable and fit for purpose. Outputs from these assessments formed a feedback loop to Stage 5 and changes were made to the compiled list.

## Results

The final outputs were (i) a COVID-19 serology minimum data standard comprised of 12 data attributes and (ii) a formalised CO-CONNECT vocabulary for laboratory analyser systems, LIMS software providers and SARS-CoV-2 serology measurements:

### COVID-19 serology minimum data standard

From the starting point of 36 data attributes from the CO-CONNECT project partners, 12 data attributes were finally considered by the group to be an absolute minimum data attribute set to allow better reporting of granular data. The minimum data standard for COVID-19 serology is shown in Table 1.

**Table 1.** COVID-19 serology minimum data standard data attributes.

Data element	Format	Value set/dictionary/definition	Group
Identifier	Varchar	NHS number (for England and Wales) or Community Health Index (CHI) number (for Scotland): this uniquely represents the person that the sample has been taken from and is being used for the test	Identifier
Date and time of test	Varchar	The date and time of when the test was performed	Test Setup
Test code (SNOMED-CT dm+d)	Varchar	Type of test being performed	
Sample type code	Varchar	Type of sample, for example, serum or plasma	
Analyser type platform/code	Varchar	Laboratory machine or method used to setup and perform the test	
Test kit name/code	Varchar	Code or name of the assay test kit used to perform the test	
Test outcome code	Varchar	Code for the outcome of test – pass or fail	Results
Qualitative result (local)	Varchar	Text result of the test – positive, negative and indeterminate	
Qualitative result value	Integer	Qualitative numerical output associated with the qualitative result	
Quantitative result value	Integer	Numerical result of the test performed – antibody binding units	
Quantitative result description	Varchar	Description of the numerical result – signal to cut-off (S/Co) ratio	
Clinical comment	Varchar	Free text comments describing the test output/outcomes	

The 12 data attributes can be grouped into 3 main areas: (a) identifier, (b) test setup and (c) results as shown in Table 1.

### Standardised vocabulary

Laboratories often used local vocabulary within the HL7 test messages. This brought about difficulties when sharing data between different laboratories or on a much grander scale, that is, nationally. Two such instances of this were the naming of laboratory analyser systems and LIMS software. No standardised naming convention (vocabulary) was observed across a randomly selected number of different laboratory result reports within England.

Thus, as part of the development work for the CSMDS, a total of 231 concepts were created to represent laboratory analyser systems (Table 2), together with 46 concepts representing numerous LIMS software (Table 3). Additionally, a further 25 concepts representing SARS-CoV-2 serology measurements for antibodies were created to formally represent both the qualitative and quantitative results produced by tests (Table 4).

**Table 2.** CO-CONNECT standardised vocabulary laboratory analyser systems concepts.

concept_name	domain_id	vocabulary_id	concept_class_id	standard_concept	concept_code	valid_start_date	valid_end_date	invalid_reason
Abbot Diagnostics ACCELERATOR a3600	Device	CO-CONNECT Analyser	Device	Null (non-standard)	CC-LAB-001	01/01/2022	31/12/2099	
Abbot Diagnostics ACCELERATOR p540	Device	CO-CONNECT Analyser	Device	Null (non-standard)	CC-LAB-002	01/01/2022	31/12/2099	
Abbot Diagnostics Alinity c	Device	CO-CONNECT Analyser	Device	Null (non-standard)	CC-LAB-003	01/01/2022	31/12/2099	
Abbot Diagnostics Alinity ci	Device	CO-CONNECT Analyser	Device	Null (non-standard)	CC-LAB-004	01/01/2022	31/12/2099	
Abbot Diagnostics Alinity hq	Device	CO-CONNECT Analyser	Device	Null (non-standard)	CC-LAB-005	01/01/2022	31/12/2099	
Abbot Diagnostics Alinity hs	Device	CO-CONNECT Analyser	Device	Null (non-standard)	CC-LAB-006	01/01/2022	31/12/2099	
Abbot Diagnostics Alinity i	Device	CO-CONNECT Analyser	Device	Null (non-standard)	CC-LAB-007	01/01/2022	31/12/2099	
Abbot Diagnostics Alinity s	Device	CO-CONNECT Analyser	Device	Null (non-standard)	CC-LAB-008	01/01/2022	31/12/2099	
Abbot Diagnostics ARCHITECT c16000	Device	CO-CONNECT Analyser	Device	Null (non-standard)	CC-LAB-009	01/01/2022	31/12/2099	
Abbot Diagnostics ARCHITECT c4000	Device	CO-CONNECT Analyser	Device	Null (non-standard)	CC-LAB-010	01/01/2022	31/12/2099	
Abbot Diagnostics ARCHITECT c8000	Device	CO-CONNECT Analyser	Device	Null (non-standard)	CC-LAB-011	01/01/2022	31/12/2099	
Abbot Diagnostics ARCHITECT i1000SR	Device	CO-CONNECT Analyser	Device	Null (non-standard)	CC-LAB-012	01/01/2022	31/12/2099	
Abbot Diagnostics ARCHITECT i2000SR	Device	CO-CONNECT Analyser	Device	Null (non-standard)	CC-LAB-013	01/01/2022	31/12/2099	
Abbot Diagnostics ARCHITECT i4000SR	Device	CO-CONNECT Analyser	Device	Null (non-standard)	CC-LAB-014	01/01/2022	31/12/2099	
Abbot Diagnostics CELL-DYN	Device	CO-CONNECT Analyser	Device	Null (non-standard)	CC-LAB-015	01/01/2022	31/12/2099	
Abbot Diagnostics CELL-DYN Emerald 22	Device	CO-CONNECT Analyser	Device	Null (non-standard)	CC-LAB-016	01/01/2022	31/12/2099	
Abbot Diagnostics CELL-DYN Emerald 22 AL	Device	CO-CONNECT Analyser	Device	Null (non-standard)	CC-LAB-017	01/01/2022	31/12/2099	
Abbot Diagnostics CELL-DYN Ruby	Device	CO-CONNECT Analyser	Device	Null (non-standard)	CC-LAB-018	01/01/2022	31/12/2099	
Abbot Diagnostics CELL-DYN Sapphire	Device	CO-CONNECT Analyser	Device	Null (non-standard)	CC-LAB-019	01/01/2022	31/12/2099	
Abbot Diagnostics GLP Systems	Device	CO-CONNECT Analyser	Device	Null (non-standard)	CC-LAB-020	01/01/2022	31/12/2099	
Beckman Coulter Access 2	Device	CO-CONNECT Analyser	Device	Null (non-standard)	CC-LAB-021	01/01/2022	31/12/2099	
Beckman Coulter APAS Independence	Device	CO-CONNECT Analyser	Device	Null (non-standard)	CC-LAB-022	01/01/2022	31/12/2099	
Beckman Coulter Aquios CL	Device	CO-CONNECT Analyser	Device	Null (non-standard)	CC-LAB-023	01/01/2022	31/12/2099	
Beckman Coulter AU 5800	Device	CO-CONNECT Analyser	Device	Null (non-standard)	CC-LAB-024	01/01/2022	31/12/2099	
Beckman Coulter AU 5810	Device	CO-CONNECT Analyser	Device	Null (non-standard)	CC-LAB-025	01/01/2022	31/12/2099	
Beckman Coulter AU 5811	Device	CO-CONNECT Analyser	Device	Null (non-standard)	CC-LAB-026	01/01/2022	31/12/2099	
Beckman Coulter AU 5812	Device	CO-CONNECT Analyser	Device	Null (non-standard)	CC-LAB-027	01/01/2022	31/12/2099	
Beckman Coulter AU 5820	Device	CO-CONNECT Analyser	Device	Null (non-standard)	CC-LAB-028	01/01/2022	31/12/2099	
Beckman Coulter AU 5821	Device	CO-CONNECT Analyser	Device	Null (non-standard)	CC-LAB-029	01/01/2022	31/12/2099	
Beckman Coulter AU 5822	Device	CO-CONNECT Analyser	Device	Null (non-standard)	CC-LAB-030	01/01/2022	31/12/2099	
Beckman Coulter AU 5830	Device	CO-CONNECT Analyser	Device	Null (non-standard)	CC-LAB-031	01/01/2022	31/12/2099	
Beckman Coulter AU 5831	Device	CO-CONNECT Analyser	Device	Null (non-standard)	CC-LAB-032	01/01/2022	31/12/2099	
Beckman Coulter AU 5832	Device	CO-CONNECT Analyser	Device	Null (non-standard)	CC-LAB-033	01/01/2022	31/12/2099	
Beckman Coulter AU 5840	Device	CO-CONNECT Analyser	Device	Null (non-standard)	CC-LAB-034	01/01/2022	31/12/2099	
Beckman Coulter AU 5841	Device	CO-CONNECT Analyser	Device	Null (non-standard)	CC-LAB-035	01/01/2022	31/12/2099	
Beckman Coulter AU 5842	Device	CO-CONNECT Analyser	Device	Null (non-standard)	CC-LAB-036	01/01/2022	31/12/2099	
Beckman Coulter AU480	Device	CO-CONNECT Analyser	Device	Null (non-standard)	CC-LAB-037	01/01/2022	31/12/2099	
Beckman Coulter Biomek 4000	Device	CO-CONNECT Analyser	Device	Null (non-standard)	CC-LAB-038	01/01/2022	31/12/2099	
Beckman Coulter Biomek i5	Device	CO-CONNECT Analyser	Device	Null (non-standard)	CC-LAB-039	01/01/2022	31/12/2099	
Beckman Coulter Biomek i7	Device	CO-CONNECT Analyser	Device	Null (non-standard)	CC-LAB-040	01/01/2022	31/12/2099	
Beckman Coulter Biomek i9	Device	CO-CONNECT Analyser	Device	Null (non-standard)	CC-LAB-041	01/01/2022	31/12/2099	
Beckman Coulter Biomek NGeniuS	Device	CO-CONNECT Analyser	Device	Null (non-standard)	CC-LAB-042	01/01/2022	31/12/2099	
Beckman Coulter DxC700 AU	Device	CO-CONNECT Analyser	Device	Null (non-standard)	CC-LAB-043	01/01/2022	31/12/2099	
Beckman Coulter DxC700 AU dTS	Device	CO-CONNECT Analyser	Device	Null (non-standard)	CC-LAB-044	01/01/2022	31/12/2099	
Beckman Coulter DxH 900	Device	CO-CONNECT Analyser	Device	Null (non-standard)	CC-LAB-045	01/01/2022	31/12/2099	
Beckman Coulter DxH 900 SMS	Device	CO-CONNECT Analyser	Device	Null (non-standard)	CC-LAB-046	01/01/2022	31/12/2099	
Beckman Coulter DxH 900-2	Device	CO-CONNECT Analyser	Device	Null (non-standard)	CC-LAB-047	01/01/2022	31/12/2099	
Beckman Coulter DxH 900-2 SMS	Device	CO-CONNECT Analyser	Device	Null (non-standard)	CC-LAB-048	01/01/2022	31/12/2099	
Beckman Coulter DxH 900-3	Device	CO-CONNECT Analyser	Device	Null (non-standard)	CC-LAB-049	01/01/2022	31/12/2099	
Beckman Coulter DxH 900-3 SMS	Device	CO-CONNECT Analyser	Device	Null (non-standard)	CC-LAB-050	01/01/2022	31/12/2099	
Beckman Coulter DxH SMS II	Device	CO-CONNECT Analyser	Device	Null (non-standard)	CC-LAB-051	01/01/2022	31/12/2099	
Beckman Coulter Dxl 1600	Device	CO-CONNECT Analyser	Device	Null (non-standard)	CC-LAB-052	01/01/2022	31/12/2099	
Beckman Coulter Dxl 600	Device	CO-CONNECT Analyser	Device	Null (non-standard)	CC-LAB-053	01/01/2022	31/12/2099	
Beckman Coulter Dxl 690T	Device	CO-CONNECT Analyser	Device	Null (non-standard)	CC-LAB-054	01/01/2022	31/12/2099	
Beckman Coulter Dxl 800	Device	CO-CONNECT Analyser	Device	Null (non-standard)	CC-LAB-055	01/01/2022	31/12/2099	
Beckman Coulter Dxl 801	Device	CO-CONNECT Analyser	Device	Null (non-standard)	CC-LAB-056	01/01/2022	31/12/2099	
Beckman Coulter DxM 1040 MicroScan WalkAway	Device	CO-CONNECT Analyser	Device	Null (non-standard)	CC-LAB-057	01/01/2022	31/12/2099	
Beckman Coulter DxM 1040 MicroScan WalkAway with LabPro	Device	CO-CONNECT Analyser	Device	Null (non-standard)	CC-LAB-058	01/01/2022	31/12/2099	
Beckman Coulter DxM 1096 MicroScan WalkAway	Device	CO-CONNECT Analyser	Device	Null (non-standard)	CC-LAB-059	01/01/2022	31/12/2099	
Beckman Coulter DxM 1096 MicroScan WalkAway with LabPro	Device	CO-CONNECT Analyser	Device	Null (non-standard)	CC-LAB-060	01/01/2022	31/12/2099	
Beckman Coulter DxU 810c Iris	Device	CO-CONNECT Analyser	Device	Null (non-standard)	CC-LAB-061	01/01/2022	31/12/2099	
Beckman Coulter DxU 840m Iris	Device	CO-CONNECT Analyser	Device	Null (non-standard)	CC-LAB-062	01/01/2022	31/12/2099	
Beckman Coulter DxU 850m Iris	Device	CO-CONNECT Analyser	Device	Null (non-standard)	CC-LAB-063	01/01/2022	31/12/2099	
Beckman Coulter DxU Iris 840 Workcell	Device	CO-CONNECT Analyser	Device	Null (non-standard)	CC-LAB-064	01/01/2022	31/12/2099	
Beckman Coulter DxU Iris 850 Workcell	Device	CO-CONNECT Analyser	Device	Null (non-standard)	CC-LAB-065	01/01/2022	31/12/2099	
Beckman Coulter IMMAGE 800 System	Device	CO-CONNECT Analyser	Device	Null (non-standard)	CC-LAB-066	01/01/2022	31/12/2099	
Beckman Coulter MicroScan AutoSCAN-4	Device	CO-CONNECT Analyser	Device	Null (non-standard)	CC-LAB-067	01/01/2022	31/12/2099	
Beckman Coulter PK7300	Device	CO-CONNECT Analyser	Device	Null (non-standard)	CC-LAB-068	01/01/2022	31/12/2099	
Beckman Coulter PK7400	Device	CO-CONNECT Analyser	Device	Null (non-standard)	CC-LAB-069	01/01/2022	31/12/2099	
Beckman Coulter WalkAway 40 plus System	Device	CO-CONNECT Analyser	Device	Null (non-standard)	CC-LAB-070	01/01/2022	31/12/2099	
Beckman Coulter WalkAway 40 plus System with LabPro	Device	CO-CONNECT Analyser	Device	Null (non-standard)	CC-LAB-071	01/01/2022	31/12/2099	
Beckman Coulter WalkAway 96 plus System	Device	CO-CONNECT Analyser	Device	Null (non-standard)	CC-LAB-072	01/01/2022	31/12/2099	
Beckman Coulter WalkAway 96 plus System with LabPro	Device	CO-CONNECT Analyser	Device	Null (non-standard)	CC-LAB-073	01/01/2022	31/12/2099	
Bio-Rad Bio-Plex 200	Device	CO-CONNECT Analyser	Device	Null (non-standard)	CC-LAB-074	01/01/2022	31/12/2099	
Bio-Rad Bio-Plex 3D	Device	CO-CONNECT Analyser	Device	Null (non-standard)	CC-LAB-075	01/01/2022	31/12/2099	
Bio-Rad ChemicDoc	Device	CO-CONNECT Analyser	Device	Null (non-standard)	CC-LAB-076	01/01/2022	31/12/2099	
Bio-Rad ChemicDoc MP	Device	CO-CONNECT Analyser	Device	Null (non-standard)	CC-LAB-077	01/01/2022	31/12/2099	
Bio-Rad ChemicDoc XRS+	Device	CO-CONNECT Analyser	Device	Null (non-standard)	CC-LAB-078	01/01/2022	31/12/2099	
Bio-Rad QX ONE Droplet Digital PCR (ddPCR) System	Device	CO-CONNECT Analyser	Device	Null (non-standard)	CC-LAB-079	01/01/2022	31/12/2099	
Bio-Rad QX200 AutoDG Droplet Digital PCR System	Device	CO-CONNECT Analyser	Device	Null (non-standard)	CC-LAB-080	01/01/2022	31/12/2099	
Bio-Rad QX200 Droplet Digital PCR (ddPCR) System	Device	CO-CONNECT Analyser	Device	Null (non-standard)	CC-LAB-081	01/01/2022	31/12/2099	
Biokit best2000	Device	CO-CONNECT Analyser	Device	Null (non-standard)	CC-LAB-082	01/01/2022	31/12/2099	
Biokit DS2	Device	CO-CONNECT Analyser	Device	Null (non-standard)	CC-LAB-083	01/01/2022	31/12/2099	
Biokit ELx50	Device	CO-CONNECT Analyser	Device	Null (non-standard)	CC-LAB-084	01/01/2022	31/12/2099	
Biokit ELx800	Device	CO-CONNECT Analyser	Device	Null (non-standard)	CC-LAB-085	01/01/2022	31/12/2099	
Biokit Monogen	Device	CO-CONNECT Analyser	Device	Null (non-standard)	CC-LAB-086	01/01/2022	31/12/2099	
bioMérieux easyMAG	Device	CO-CONNECT Analyser	Device	Null (non-standard)	CC-LAB-087	01/01/2022	31/12/2099	
bioMérieux EMAG	Device	CO-CONNECT Analyser	Device	Null (non-standard)	CC-LAB-088	01/01/2022	31/12/2099	
Diasorin ETI-Max 3000	Device	CO-CONNECT Analyser	Device	Null (non-standard)	CC-LAB-089	01/01/2022	31/12/2099	
Diasorin LIAISON	Device	CO-CONNECT Analyser	Device	Null (non-standard)	CC-LAB-090	01/01/2022	31/12/2099	
Diasorin LIAISON XL	Device	CO-CONNECT Analyser	Device	Null (non-standard)	CC-LAB-091	01/01/2022	31/12/2099	
Diasorin LIAISON XS	Device	CO-CONNECT Analyser	Device	Null (non-standard)	CC-LAB-092	01/01/2022	31/12/2099	
Dynex Agility	Device	CO-CONNECT Analyser	Device	Null (non-standard)	CC-LAB-093	01/01/2022	31/12/2099	
Dynex DS2	Device	CO-CONNECT Analyser	Device	Null (non-standard)	CC-LAB-094	01/01/2022	31/12/2099	
Dynex DSX	Device	CO-CONNECT Analyser	Device	Null (non-standard)	CC-LAB-095	01/01/2022	31/12/2099	
Dynex Multiplier	Device	CO-CONNECT Analyser	Device	Null (non-standard)	CC-LAB-096	01/01/2022	31/12/2099	
Euroimmun Analyser I	Device	CO-CONNECT Analyser	Device	Null (non-standard)	CC-LAB-097	01/01/2022	31/12/2099	
Euroimmun Analyser I-2P	Device	CO-CONNECT Analyser	Device	Null (non-standard)	CC-LAB-098	01/01/2022	31/12/2099	
Euroimmun EUROArrayScanner	Device	CO-CONNECT Analyser	Device	Null (non-standard)	CC-LAB-099	01/01/2022	31/12/2099	
Euroimmun EUROBlotMaster	Device	CO-CONNECT Analyser	Device	Null (non-standard)	CC-LAB-100	01/01/2022	31/12/2099	
Euroimmun EUROBlotOne	Device	CO-CONNECT Analyser	Device	Null (non-standard)	CC-LAB-101	01/01/2022	31/12/2099	
Euroimmun EUROLabWorkstation ELISA	Device	CO-CONNECT Analyser	Device	Null (non-standard)	CC-LAB-102	01/01/2022	31/12/2099	
Euroimmun EUROLabWorkstation IFA	Device	CO-CONNECT Analyser	Device	Null (non-standard)	CC-LAB-103	01/01/2022	31/12/2099	
Euroimmun EUROPattern	Device	CO-CONNECT Analyser	Device	Null (non-standard)	CC-LAB-104	01/01/2022	31/12/2099	
Euroimmun EUROPattern Microscope Live	Device	CO-CONNECT Analyser	Device	Null (non-standard)	CC-LAB-105	01/01/2022	31/12/2099	
Euroimmun EUROStar III Plus	Device	CO-CONNECT Analyser	Device	Null (non-standard)	CC-LAB-106	01/01/2022	31/12/2099	
Euroimmun IF Sprinter	Device	CO-CONNECT Analyser	Device	Null (non-standard)	CC-LAB-107	01/01/2022	31/12/2099	
Euroimmun MERGITE!	Device	CO-CONNECT Analyser	Device	Null (non-standard)	CC-LAB-108	01/01/2022	31/12/2099	
Euroimmun Pre-NAT II	Device	CO-CONNECT Analyser	Device	Null (non-standard)	CC-LAB-109	01/01/2022	31/12/2099	
Euroimmun RA Analyser 10	Device	CO-CONNECT Analyser	Device	Null (non-standard)	CC-LAB-110	01/01/2022	31/12/2099	
Euroimmun Sprinter XL	Device	CO-CONNECT Analyser	Device	Null (non-standard)	CC-LAB-111	01/01/2022	31/12/2099	
Grifols Chorus Trio	Device	CO-CONNECT Analyser	Device	Null (non-standard)	CC-LAB-112	01/01/2022	31/12/2099	
Grifols HELIOS	Device	CO-CONNECT Analyser	Device	Null (non-standard)	CC-LAB-113	01/01/2022	31/12/2099	
Grifols HELMED	Device	CO-CONNECT Analyser	Device	Null (non-standard)	CC-LAB-114	01/01/2022	31/12/2099	
Grifols SQII ELISA	Device	CO-CONNECT Analyser	Device	Null (non-standard)	CC-LAB-115	01/01/2022	31/12/2099	
Grifols Triturus ELISA	Device	CO-CONNECT Analyser	Device	Null (non-standard)	CC-LAB-116	01/01/2022	31/12/2099	
Immucor Echo Lumenas	Device	CO-CONNECT Analyser	Device	Null (non-standard)	CC-LAB-117	01/01/2022	31/12/2099	
Immucor NEO	Device	CO-CONNECT Analyser	Device	Null (non-standard)	CC-LAB-118	01/01/2022	31/12/2099	
Ortho Clinical Diagnostics Vitros 3600	Device	CO-CONNECT Analyser	Device	Null (non-standard)	CC-LAB-119	01/01/2022	31/12/2099	
Ortho Clinical Diagnostics Vitros 4600	Device	CO-CONNECT Analyser	Device	Null (non-standard)	CC-LAB-120	01/01/2022	31/12/2099	
Ortho Clinical Diagnostics Vitros 5600	Device	CO-CONNECT Analyser	Device	Null (non-standard)	CC-LAB-121	01/01/2022	31/12/2099	
Ortho Clinical Diagnostics Vitros ECiQ	Device	CO-CONNECT Analyser	Device	Null (non-standard)	CC-LAB-122	01/01/2022	31/12/2099	
Ortho Clinical Diagnostics Vitros XT 3400	Device	CO-CONNECT Analyser	Device	Null (non-standard)	CC-LAB-123	01/01/2022	31/12/2099	
Ortho Clinical Diagnostics Vitros XT 7600	Device	CO-CONNECT Analyser	Device	Null (non-standard)	CC-LAB-124	01/01/2022	31/12/2099	
Roche Cobas 4000	Device	CO-CONNECT Analyser	Device	Null (non-standard)	CC-LAB-125	01/01/2022	31/12/2099	
Roche Cobas 4000 c311	Device	CO-CONNECT Analyser	Device	Null (non-standard)	CC-LAB-126	01/01/2022	31/12/2099	
Roche Cobas 4000 e411	Device	CO-CONNECT Analyser	Device	Null (non-standard)	CC-LAB-127	01/01/2022	31/12/2099	
Roche Cobas 6000	Device	CO-CONNECT Analyser	Device	Null (non-standard)	CC-LAB-128	01/01/2022	31/12/2099	
Roche Cobas 6000 c501	Device	CO-CONNECT Analyser	Device	Null (non-standard)	CC-LAB-129	01/01/2022	31/12/2099	
Roche Cobas 6800	Device	CO-CONNECT Analyser	Device	Null (non-standard)	CC-LAB-130	01/01/2022	31/12/2099	
Roche Cobas 8000	Device	CO-CONNECT Analyser	Device	Null (non-standard)	CC-LAB-131	01/01/2022	31/12/2099	
Roche Cobas 8000 c502	Device	CO-CONNECT Analyser	Device	Null (non-standard)	CC-LAB-132	01/01/2022	31/12/2099	
Roche Cobas 8000 c701	Device	CO-CONNECT Analyser	Device	Null (non-standard)	CC-LAB-133	01/01/2022	31/12/2099	
Roche Cobas 8000 c702	Device	CO-CONNECT Analyser	Device	Null (non-standard)	CC-LAB-134	01/01/2022	31/12/2099	
Roche Cobas 8800	Device	CO-CONNECT Analyser	Device	Null (non-standard)	CC-LAB-135	01/01/2022	31/12/2099	
Sebia Capillary 2	Device	CO-CONNECT Analyser	Device	Null (non-standard)	CC-LAB-136	01/01/2022	31/12/2099	
Sebia Capillarys 3	Device	CO-CONNECT Analyser	Device	Null (non-standard)	CC-LAB-137	01/01/2022	31/12/2099	
Sebia Capillarys 3 OCTA	Device	CO-CONNECT Analyser	Device	Null (non-standard)	CC-LAB-138	01/01/2022	31/12/2099	
Sebia Capillarys 3 TERA	Device	CO-CONNECT Analyser	Device	Null (non-standard)	CC-LAB-139	01/01/2022	31/12/2099	
Sebia Capillarys 3 TERA MC	Device	CO-CONNECT Analyser	Device	Null (non-standard)	CC-LAB-140	01/01/2022	31/12/2099	
Sebia Capillarys 3 TERA TLA	Device	CO-CONNECT Analyser	Device	Null (non-standard)	CC-LAB-141	01/01/2022	31/12/2099	
Sebia Hydrasys 2	Device	CO-CONNECT Analyser	Device	Null (non-standard)	CC-LAB-142	01/01/2022	31/12/2099	
Sebia Minicap Flex-Piercing	Device	CO-CONNECT Analyser	Device	Null (non-standard)	CC-LAB-143	01/01/2022	31/12/2099	
Siemens ADIVA 1240 Chemistry System	Device	CO-CONNECT Analyser	Device	Null (non-standard)	CC-LAB-144	01/01/2022	31/12/2099	
Siemens ADIVA 1800 Chemistry System	Device	CO-CONNECT Analyser	Device	Null (non-standard)	CC-LAB-145	01/01/2022	31/12/2099	
Siemens ADIVA Chemistry XPT System	Device	CO-CONNECT Analyser	Device	Null (non-standard)	CC-LAB-146	01/01/2022	31/12/2099	
Siemens ADVIA Centaur CP Immunoassay System	Device	CO-CONNECT Analyser	Device	Null (non-standard)	CC-LAB-147	01/01/2022	31/12/2099	
Siemens ADVIA Centaur XP Immunoassay System	Device	CO-CONNECT Analyser	Device	Null (non-standard)	CC-LAB-148	01/01/2022	31/12/2099	
Siemens ADVIA Centaur XPT Immunoassay System	Device	CO-CONNECT Analyser	Device	Null (non-standard)	CC-LAB-149	01/01/2022	31/12/2099	
Siemens Atellica	Device	CO-CONNECT Analyser	Device	Null (non-standard)	CC-LAB-150	01/01/2022	31/12/2099	
Siemens Atellica 1500	Device	CO-CONNECT Analyser	Device	Null (non-standard)	CC-LAB-151	01/01/2022	31/12/2099	
Siemens Atellica CH 930	Device	CO-CONNECT Analyser	Device	Null (non-standard)	CC-LAB-152	01/01/2022	31/12/2099	
Siemens Atellica IM 1300	Device	CO-CONNECT Analyser	Device	Null (non-standard)	CC-LAB-153	01/01/2022	31/12/2099	
Siemens Atellica IM 1600	Device	CO-CONNECT Analyser	Device	Null (non-standard)	CC-LAB-154	01/01/2022	31/12/2099	
Siemens Atellica NEPH 630 System	Device	CO-CONNECT Analyser	Device	Null (non-standard)	CC-LAB-155	01/01/2022	31/12/2099	
Siemens IMMUlITE 2000XPi Immunoassay System	Device	CO-CONNECT Analyser	Device	Null (non-standard)	CC-LAB-156	01/01/2022	31/12/2099	
STAGO Compact Max	Device	CO-CONNECT Analyser	Device	Null (non-standard)	CC-LAB-157	01/01/2022	31/12/2099	
STAGO Compact Max 3	Device	CO-CONNECT Analyser	Device	Null (non-standard)	CC-LAB-158	01/01/2022	31/12/2099	
STAGO ST Genesia System	Device	CO-CONNECT Analyser	Device	Null (non-standard)	CC-LAB-159	01/01/2022	31/12/2099	
STAGO STA Satellite Max	Device	CO-CONNECT Analyser	Device	Null (non-standard)	CC-LAB-160	01/01/2022	31/12/2099	
STAGO STA-R Max	Device	CO-CONNECT Analyser	Device	Null (non-standard)	CC-LAB-161	01/01/2022	31/12/2099	
STAGO STA-R Max 3	Device	CO-CONNECT Analyser	Device	Null (non-standard)	CC-LAB-162	01/01/2022	31/12/2099	
STAGO STart Max	Device	CO-CONNECT Analyser	Device	Null (non-standard)	CC-LAB-163	01/01/2022	31/12/2099	
Starrsed AutoCompact analyser	Device	CO-CONNECT Analyser	Device	Null (non-standard)	CC-LAB-164	01/01/2022	31/12/2099	
Starrsed InteRRLiner	Device	CO-CONNECT Analyser	Device	Null (non-standard)	CC-LAB-165	01/01/2022	31/12/2099	
Starrsed RL	Device	CO-CONNECT Analyser	Device	Null (non-standard)	CC-LAB-166	01/01/2022	31/12/2099	
Starrsed RS	Device	CO-CONNECT Analyser	Device	Null (non-standard)	CC-LAB-167	01/01/2022	31/12/2099	
Starrsed ST	Device	CO-CONNECT Analyser	Device	Null (non-standard)	CC-LAB-168	01/01/2022	31/12/2099	
Starrsed TL	Device	CO-CONNECT Analyser	Device	Null (non-standard)	CC-LAB-169	01/01/2022	31/12/2099	
Sysmex Cellavision DC-1	Device	CO-CONNECT Analyser	Device	Null (non-standard)	CC-LAB-170	01/01/2022	31/12/2099	
Sysmex Cellavision DM1200	Device	CO-CONNECT Analyser	Device	Null (non-standard)	CC-LAB-171	01/01/2022	31/12/2099	
Sysmex CN-6000	Device	CO-CONNECT Analyser	Device	Null (non-standard)	CC-LAB-172	01/01/2022	31/12/2099	
Sysmex Cyflow Cube 6	Device	CO-CONNECT Analyser	Device	Null (non-standard)	CC-LAB-173	01/01/2022	31/12/2099	
Sysmex Cyflow Cube 8	Device	CO-CONNECT Analyser	Device	Null (non-standard)	CC-LAB-174	01/01/2022	31/12/2099	
Sysmex Cyflow Polidy	Device	CO-CONNECT Analyser	Device	Null (non-standard)	CC-LAB-175	01/01/2022	31/12/2099	
Sysmex Cyflow Space	Device	CO-CONNECT Analyser	Device	Null (non-standard)	CC-LAB-176	01/01/2022	31/12/2099	
Sysmex DI-60	Device	CO-CONNECT Analyser	Device	Null (non-standard)	CC-LAB-177	01/01/2022	31/12/2099	
Sysmex OSNA	Device	CO-CONNECT Analyser	Device	Null (non-standard)	CC-LAB-178	01/01/2022	31/12/2099	
Sysmex PS-10	Device	CO-CONNECT Analyser	Device	Null (non-standard)	CC-LAB-179	01/01/2022	31/12/2099	
Sysmex SENTIFIT 270	Device	CO-CONNECT Analyser	Device	Null (non-standard)	CC-LAB-180	01/01/2022	31/12/2099	
Sysmex SP-10	Device	CO-CONNECT Analyser	Device	Null (non-standard)	CC-LAB-181	01/01/2022	31/12/2099	
Sysmex TOSOH G11	Device	CO-CONNECT Analyser	Device	Null (non-standard)	CC-LAB-182	01/01/2022	31/12/2099	
Sysmex UC-3500	Device	CO-CONNECT Analyser	Device	Null (non-standard)	CC-LAB-183	01/01/2022	31/12/2099	
Sysmex UD-10	Device	CO-CONNECT Analyser	Device	Null (non-standard)	CC-LAB-184	01/01/2022	31/12/2099	
Sysmex UF-5000	Device	CO-CONNECT Analyser	Device	Null (non-standard)	CC-LAB-185	01/01/2022	31/12/2099	
Sysmex XE-2100	Device	CO-CONNECT Analyser	Device	Null (non-standard)	CC-LAB-186	01/01/2022	31/12/2099	
Sysmex XE-5000	Device	CO-CONNECT Analyser	Device	Null (non-standard)	CC-LAB-187	01/01/2022	31/12/2099	
Sysmex XN-1000	Device	CO-CONNECT Analyser	Device	Null (non-standard)	CC-LAB-188	01/01/2022	31/12/2099	
Sysmex XN-1000 PURE	Device	CO-CONNECT Analyser	Device	Null (non-standard)	CC-LAB-189	01/01/2022	31/12/2099	
Sysmex XN-1000V	Device	CO-CONNECT Analyser	Device	Null (non-standard)	CC-LAB-190	01/01/2022	31/12/2099	
Sysmex XN-1500	Device	CO-CONNECT Analyser	Device	Null (non-standard)	CC-LAB-191	01/01/2022	31/12/2099	
Sysmex XN-2000	Device	CO-CONNECT Analyser	Device	Null (non-standard)	CC-LAB-192	01/01/2022	31/12/2099	
Sysmex XN-2000V	Device	CO-CONNECT Analyser	Device	Null (non-standard)	CC-LAB-193	01/01/2022	31/12/2099	
Sysmex XN-300	Device	CO-CONNECT Analyser	Device	Null (non-standard)	CC-LAB-194	01/01/2022	31/12/2099	
Sysmex XN-3000	Device	CO-CONNECT Analyser	Device	Null (non-standard)	CC-LAB-195	01/01/2022	31/12/2099	
Sysmex XN-3100	Device	CO-CONNECT Analyser	Device	Null (non-standard)	CC-LAB-196	01/01/2022	31/12/2099	
Sysmex XN-350	Device	CO-CONNECT Analyser	Device	Null (non-standard)	CC-LAB-197	01/01/2022	31/12/2099	
Sysmex XN-450	Device	CO-CONNECT Analyser	Device	Null (non-standard)	CC-LAB-198	01/01/2022	31/12/2099	
Sysmex XN-550	Device	CO-CONNECT Analyser	Device	Null (non-standard)	CC-LAB-199	01/01/2022	31/12/2099	
Sysmex XN-9000	Device	CO-CONNECT Analyser	Device	Null (non-standard)	CC-LAB-200	01/01/2022	31/12/2099	
Sysmex XN-9100	Device	CO-CONNECT Analyser	Device	Null (non-standard)	CC-LAB-201	01/01/2022	31/12/2099	
Sysmex XN-9100 Compact Integration	Device	CO-CONNECT Analyser	Device	Null (non-standard)	CC-LAB-202	01/01/2022	31/12/2099	
Sysmex XN-9100 Maximum Workload	Device	CO-CONNECT Analyser	Device	Null (non-standard)	CC-LAB-203	01/01/2022	31/12/2099	
Sysmex XN-9100 Sorting & Archiving	Device	CO-CONNECT Analyser	Device	Null (non-standard)	CC-LAB-204	01/01/2022	31/12/2099	
Sysmex XN-9100 Workload Balance	Device	CO-CONNECT Analyser	Device	Null (non-standard)	CC-LAB-205	01/01/2022	31/12/2099	
Sysmex XP-300	Device	CO-CONNECT Analyser	Device	Null (non-standard)	CC-LAB-206	01/01/2022	31/12/2099	
TECAN D300e Digital Dispenser	Device	CO-CONNECT Analyser	Device	Null (non-standard)	CC-LAB-207	01/01/2022	31/12/2099	
TECAN Fluent	Device	CO-CONNECT Analyser	Device	Null (non-standard)	CC-LAB-208	01/01/2022	31/12/2099	
TECAN Fluent Automation Workstation	Device	CO-CONNECT Analyser	Device	Null (non-standard)	CC-LAB-209	01/01/2022	31/12/2099	
TECAN Freedom	Device	CO-CONNECT Analyser	Device	Null (non-standard)	CC-LAB-210	01/01/2022	31/12/2099	
TECAN Freedom EVO	Device	CO-CONNECT Analyser	Device	Null (non-standard)	CC-LAB-211	01/01/2022	31/12/2099	
TECAN Hydro	Device	CO-CONNECT Analyser	Device	Null (non-standard)	CC-LAB-212	01/01/2022	31/12/2099	
TECAN HydroFlex	Device	CO-CONNECT Analyser	Device	Null (non-standard)	CC-LAB-213	01/01/2022	31/12/2099	
TECAN HydroSpeed	Device	CO-CONNECT Analyser	Device	Null (non-standard)	CC-LAB-214	01/01/2022	31/12/2099	
TECAN Infinite	Device	CO-CONNECT Analyser	Device	Null (non-standard)	CC-LAB-215	01/01/2022	31/12/2099	
TECAN Infinite 200 PRO	Device	CO-CONNECT Analyser	Device	Null (non-standard)	CC-LAB-216	01/01/2022	31/12/2099	
TECAN Infinite F50	Device	CO-CONNECT Analyser	Device	Null (non-standard)	CC-LAB-217	01/01/2022	31/12/2099	
TECAN Infinite F50 Robotoic	Device	CO-CONNECT Analyser	Device	Null (non-standard)	CC-LAB-218	01/01/2022	31/12/2099	
TECAN LABWERX	Device	CO-CONNECT Analyser	Device	Null (non-standard)	CC-LAB-219	01/01/2022	31/12/2099	
TECAN Lumi	Device	CO-CONNECT Analyser	Device	Null (non-standard)	CC-LAB-220	01/01/2022	31/12/2099	
TECAN M Nano	Device	CO-CONNECT Analyser	Device	Null (non-standard)	CC-LAB-221	01/01/2022	31/12/2099	
TECAN Spark	Device	CO-CONNECT Analyser	Device	Null (non-standard)	CC-LAB-222	01/01/2022	31/12/2099	
TECAN Spark Cyto	Device	CO-CONNECT Analyser	Device	Null (non-standard)	CC-LAB-223	01/01/2022	31/12/2099	
TECAN Sunrise	Device	CO-CONNECT Analyser	Device	Null (non-standard)	CC-LAB-224	01/01/2022	31/12/2099	
Trinity Biotech DS2	Device	CO-CONNECT Analyser	Device	Null (non-standard)	CC-LAB-225	01/01/2022	31/12/2099	
Trinity Biotech DSX	Device	CO-CONNECT Analyser	Device	Null (non-standard)	CC-LAB-226	01/01/2022	31/12/2099	
Trinity Biotech Fluorescence	Device	CO-CONNECT Analyser	Device	Null (non-standard)	CC-LAB-227	01/01/2022	31/12/2099	
Trinity Biotech Premier Hb9210	Device	CO-CONNECT Analyser	Device	Null (non-standard)	CC-LAB-228	01/01/2022	31/12/2099	
Trinity Biotech Tri-stat	Device	CO-CONNECT Analyser	Device	Null (non-standard)	CC-LAB-229	01/01/2022	31/12/2099	
Trinity Biotech Tri-stat 2	Device	CO-CONNECT Analyser	Device	Null (non-standard)	CC-LAB-230	01/01/2022	31/12/2099	
Vitech V8 Interliner a	Device	CO-CONNECT Analyser	Device	Null (non-standard)	CC-LAB-231	01/01/2022	31/12/2099

**Table 3.** CO-CONNECT standardised vocabulary LIMS software concepts.

concept_name	domain_id	vocabulary_id	concept_class_id	standard_concept	concept_code	valid_start_date	valid_end_date	invalid_reason
Agilent SLIMS	Device	CO-CONNECT LIMS-LIS	Device	Null (non-standard)	CC-LIMS-001	01/01/2022	31/12/2099	
ApexHealthCare Apex LIS	Device	CO-CONNECT LIMS-LIS	Device	Null (non-standard)	CC-LIMS-002	01/01/2022	31/12/2099	
Autoscribe Informatics COVID LIMS	Device	CO-CONNECT LIMS-LIS	Device	Null (non-standard)	CC-LIMS-003	01/01/2022	31/12/2099	
Autoscribe Informatics Express	Device	CO-CONNECT LIMS-LIS	Device	Null (non-standard)	CC-LIMS-004	01/01/2022	31/12/2099	
Autoscribe Informatics Gemini	Device	CO-CONNECT LIMS-LIS	Device	Null (non-standard)	CC-LIMS-005	01/01/2022	31/12/2099	
Autoscribe Informatics Stability	Device	CO-CONNECT LIMS-LIS	Device	Null (non-standard)	CC-LIMS-006	01/01/2022	31/12/2099	
Autoscribe Informatics Tracker	Device	CO-CONNECT LIMS-LIS	Device	Null (non-standard)	CC-LIMS-007	01/01/2022	31/12/2099	
Benchling LIMS	Device	CO-CONNECT LIMS-LIS	Device	Null (non-standard)	CC-LIMS-008	01/01/2022	31/12/2099	
CGM LABDAQ	Device	CO-CONNECT LIMS-LIS	Device	Null (non-standard)	CC-LIMS-009	01/01/2022	31/12/2099	
Cirdan Ultra	Device	CO-CONNECT LIMS-LIS	Device	Null (non-standard)	CC-LIMS-010	01/01/2022	31/12/2099	
Clinisys LabCentre	Device	CO-CONNECT LIMS-LIS	Device	Null (non-standard)	CC-LIMS-011	01/01/2022	31/12/2099	
Clinisys WinPath	Device	CO-CONNECT LIMS-LIS	Device	Null (non-standard)	CC-LIMS-012	01/01/2022	31/12/2099	
CloudLIMS	Device	CO-CONNECT LIMS-LIS	Device	Null (non-standard)	CC-LIMS-013	01/01/2022	31/12/2099	
Computer Frameworks Lab Management System	Device	CO-CONNECT LIMS-LIS	Device	Null (non-standard)	CC-LIMS-014	01/01/2022	31/12/2099	
CrelioHealth LIMS	Device	CO-CONNECT LIMS-LIS	Device	Null (non-standard)	CC-LIMS-015	01/01/2022	31/12/2099	
Dendi Software Dendi LIS	Device	CO-CONNECT LIMS-LIS	Device	Null (non-standard)	CC-LIMS-016	01/01/2022	31/12/2099	
DXC Apex	Device	CO-CONNECT LIMS-LIS	Device	Null (non-standard)	CC-LIMS-017	01/01/2022	31/12/2099	
DXC Telepath	Device	CO-CONNECT LIMS-LIS	Device	Null (non-standard)	CC-LIMS-018	01/01/2022	31/12/2099	
Epic Beaker	Device	CO-CONNECT LIMS-LIS	Device	Null (non-standard)	CC-LIMS-019	01/01/2022	31/12/2099	
Labgen LIS	Device	CO-CONNECT LIMS-LIS	Device	Null (non-standard)	CC-LIMS-020	01/01/2022	31/12/2099	
Labguru LIMs	Device	CO-CONNECT LIMS-LIS	Device	Null (non-standard)	CC-LIMS-021	01/01/2022	31/12/2099	
LabTrak LIS	Device	CO-CONNECT LIMS-LIS	Device	Null (non-standard)	CC-LIMS-022	01/01/2022	31/12/2099	
LabVantage LIMS	Device	CO-CONNECT LIMS-LIS	Device	Null (non-standard)	CC-LIMS-023	01/01/2022	31/12/2099	
LabWare LIMS	Device	CO-CONNECT LIMS-LIS	Device	Null (non-standard)	CC-LIMS-024	01/01/2022	31/12/2099	
LigoLab liS and RCM	Device	CO-CONNECT LIMS-LIS	Device	Null (non-standard)	CC-LIMS-025	01/01/2022	31/12/2099	
Mak-System e-Traceline	Device	CO-CONNECT LIMS-LIS	Device	Null (non-standard)	CC-LIMS-026	01/01/2022	31/12/2099	
MBioLIMS BioBanking	Device	CO-CONNECT LIMS-LIS	Device	Null (non-standard)	CC-LIMS-027	01/01/2022	31/12/2099	
Mill Systems MillCare	Device	CO-CONNECT LIMS-LIS	Device	Null (non-standard)	CC-LIMS-028	01/01/2022	31/12/2099	
NHS Custom Built LIMS	Device	CO-CONNECT LIMS-LIS	Device	Null (non-standard)	CC-LIMS-029	01/01/2022	31/12/2099	
Onlims Online LIMS	Device	CO-CONNECT LIMS-LIS	Device	Null (non-standard)	CC-LIMS-030	01/01/2022	31/12/2099	
SciNote	Device	CO-CONNECT LIMS-LIS	Device	Null (non-standard)	CC-LIMS-031	01/01/2022	31/12/2099	
Siemens Atellica Data Manager	Device	CO-CONNECT LIMS-LIS	Device	Null (non-standard)	CC-LIMS-032	01/01/2022	31/12/2099	
Siemens Atellica Process Manager	Device	CO-CONNECT LIMS-LIS	Device	Null (non-standard)	CC-LIMS-033	01/01/2022	31/12/2099	
Soft Computer SoftLab	Device	CO-CONNECT LIMS-LIS	Device	Null (non-standard)	CC-LIMS-034	01/01/2022	31/12/2099	
STARLIMS	Device	CO-CONNECT LIMS-LIS	Device	Null (non-standard)	CC-LIMS-035	01/01/2022	31/12/2099	
Sunquest Laboratory	Device	CO-CONNECT LIMS-LIS	Device	Null (non-standard)	CC-LIMS-036	01/01/2022	31/12/2099	
Sussex Biologicals Bank Manager	Device	CO-CONNECT LIMS-LIS	Device	Null (non-standard)	CC-LIMS-037	01/01/2022	31/12/2099	
Sysmex Delphic LIS	Device	CO-CONNECT LIMS-LIS	Device	Null (non-standard)	CC-LIMS-038	01/01/2022	31/12/2099	
Technidata BactiLink	Device	CO-CONNECT LIMS-LIS	Device	Null (non-standard)	CC-LIMS-039	01/01/2022	31/12/2099	
Technidata Genet	Device	CO-CONNECT LIMS-LIS	Device	Null (non-standard)	CC-LIMS-040	01/01/2022	31/12/2099	
Technidata HistoCyto	Device	CO-CONNECT LIMS-LIS	Device	Null (non-standard)	CC-LIMS-041	01/01/2022	31/12/2099	
Technidata NexLabs	Device	CO-CONNECT LIMS-LIS	Device	Null (non-standard)	CC-LIMS-042	01/01/2022	31/12/2099	
Technidata Workstation	Device	CO-CONNECT LIMS-LIS	Device	Null (non-standard)	CC-LIMS-043	01/01/2022	31/12/2099	
Thermo Fishcer Sample Manager	Device	CO-CONNECT LIMS-LIS	Device	Null (non-standard)	CC-LIMS-044	01/01/2022	31/12/2099	
ThirdWave Analytics Lockbox LIMS	Device	CO-CONNECT LIMS-LIS	Device	Null (non-standard)	CC-LIMS-045	01/01/2022	31/12/2099	
Well Sky	Device	CO-CONNECT LIMS-LIS	Device	Null (non-standard)	CC-LIMS-046	01/01/2022	31/12/2099

**Table 4.** CO-CONNECT standardised vocabulary SARS-COV-2 serology measurement concepts.

concept_name	domain_id	vocabulary_id	concept_class_id	standard_concept	concept_code	valid_start_date	valid_end_date	invalid_reason
SARS-CoV-2 antibody detection result positive	Measurement	CO-CONNECT Serology	precoordinated pair	Null (non-standard)	CC-SEROLOGY-001	01/01/2022	31/12/2099	
SARS-CoV-2 antibody detection result negative	Measurement	CO-CONNECT Serology	precoordinated pair	Null (non-standard)	CC-SEROLOGY-002	01/01/2022	31/12/2099	
SARS-CoV-2 antibody detection result indeterminate	Measurement	CO-CONNECT Serology	precoordinated pair	Null (non-standard)	CC-SEROLOGY-003	01/01/2022	31/12/2099	
SARS-CoV-2 IgA antibody detection result positive	Measurement	CO-CONNECT Serology	precoordinated pair	Null (non-standard)	CC-SEROLOGY-004	01/01/2022	31/12/2099	
SARS-CoV-2 IgA antibody detection result negative	Measurement	CO-CONNECT Serology	precoordinated pair	Null (non-standard)	CC-SEROLOGY-005	01/01/2022	31/12/2099	
SARS-CoV-2 IgA antibody detection result indeterminate	Measurement	CO-CONNECT Serology	precoordinated pair	Null (non-standard)	CC-SEROLOGY-006	01/01/2022	31/12/2099	
SARS-CoV-2 IgM antibody detection result positive	Measurement	CO-CONNECT Serology	precoordinated pair	Null (non-standard)	CC-SEROLOGY-007	01/01/2022	31/12/2099	
SARS-CoV-2 IgM antibody detection result negative	Measurement	CO-CONNECT Serology	precoordinated pair	Null (non-standard)	CC-SEROLOGY-008	01/01/2022	31/12/2099	
SARS-CoV-2 IgM antibody detection result indeterminate	Measurement	CO-CONNECT Serology	precoordinated pair	Null (non-standard)	CC-SEROLOGY-009	01/01/2022	31/12/2099	
SARS-CoV-2 IgG antibody detection result positive	Measurement	CO-CONNECT Serology	precoordinated pair	Null (non-standard)	CC-SEROLOGY-010	01/01/2022	31/12/2099	
SARS-CoV-2 IgG antibody detection result negative	Measurement	CO-CONNECT Serology	precoordinated pair	Null (non-standard)	CC-SEROLOGY-011	01/01/2022	31/12/2099	
SARS-CoV-2 IgG antibody detection result indeterminate	Measurement	CO-CONNECT Serology	precoordinated pair	Null (non-standard)	CC-SEROLOGY-012	01/01/2022	31/12/2099	
SARS-CoV-2 IgM + IgG antibody detection result positive	Measurement	CO-CONNECT Serology	precoordinated pair	Null (non-standard)	CC-SEROLOGY-013	01/01/2022	31/12/2099	
SARS-CoV-2 IgM + IgG antibody detection result negative	Measurement	CO-CONNECT Serology	precoordinated pair	Null (non-standard)	CC-SEROLOGY-014	01/01/2022	31/12/2099	
SARS-CoV-2 IgM + IgG antibody detection result indeterminate	Measurement	CO-CONNECT Serology	precoordinated pair	Null (non-standard)	CC-SEROLOGY-015	01/01/2022	31/12/2099	
Measurement of severe acute respiratory syndrome coronavirus-2 antibody IgA	Measurement	CO-CONNECT Serology	Measurement	Null (non-standard)	CC-SEROLOGY-016	01/01/2022	31/12/2099	
Measurement of severe acute respiratory syndrome coronavirus-2 antibody IgM	Measurement	CO-CONNECT Serology	Measurement	Null (non-standard)	CC-SEROLOGY-017	01/01/2022	31/12/2099	
Measurement of severe acute respiratory syndrome coronavirus-2 antibody IgG	Measurement	CO-CONNECT Serology	Measurement	Null (non-standard)	CC-SEROLOGY-018	01/01/2022	31/12/2099	
Measurement of severe acute respiratory syndrome coronavirus-2 antibody IgM+IgG	Measurement	CO-CONNECT Serology	Measurement	Null (non-standard)	CC-SEROLOGY-019	01/01/2022	31/12/2099	
SARS-CoV-2 antibody to nucleocapsid (N) protein present	Measurement	CO-CONNECT Serology	precoordinated pair	Null (non-standard)	CC-SEROLOGY-020	01/01/2022	31/12/2099	
SARS-CoV-2 antibody to spike (S) protein present	Measurement	CO-CONNECT Serology	precoordinated pair	Null (non-standard)	CC-SEROLOGY-021	01/01/2022	31/12/2099	
SARS-CoV-2 antibody to nucleocapsid (N) protein absent	Measurement	CO-CONNECT Serology	precoordinated pair	Null (non-standard)	CC-SEROLOGY-022	01/01/2022	31/12/2099	
SARS-CoV-2 antibody to spike (S) protein absent	Measurement	CO-CONNECT Serology	precoordinated pair	Null (non-standard)	CC-SEROLOGY-023	01/01/2022	31/12/2099	
SARS-CoV-2 antibody to nucleocapsid (N) protein indeterminate	Measurement	CO-CONNECT Serology	precoordinated pair	Null (non-standard)	CC-SEROLOGY-024	01/01/2022	31/12/2099	
SARS-CoV-2 antibody to spike (S) protein indeterminate	Measurement	CO-CONNECT Serology	precoordinated pair	Null (non-standard)	CC-SEROLOGY-025	01/01/2022	31/12/2099	

The Observational Medical Outcomes Partnership (OMOP) Common Data Model (CDM) approach to the description of concepts was utilised to structure the expression of the CO-CONNECT vocabulary concepts.^72,73^ Employing the OMOP CDM schema to describe concepts in a standardised manner supports interoperability and reuse.^74–77^ Tables 2–4 illustrate how the concepts were formalised by IQVIA (a company who provide OHDSI and OMOP services) and form part of the larger standardised CO-CONNECT vocabulary that has been published for universal use.

Each concept of the CO-CONNECT vocabulary has a ‘concept_name’ and a ‘domain_id’ (a unique domain identification). The laboratory analyser systems and LIMS software have a domain_id of device (see Tables 2 and 3), whilst the SARS-CoV-2 serology measurement domain_id is a measurement (see Table 4). The ‘vocabulary_id’ is a code that represents each vocabulary. For the laboratory analyser systems it is CO-CONNECT Analyser, for LIMS software it is CO-CONNECT LIMS-LIS and for the SARS-CoV-2 serology measurements it is CO-CONNECT Serology. The ‘concept_class_id’ is a semantic tag which is a unique identifier for the class a specific concept belongs to.^74^ The concept_class_id for laboratory analyser systems (see Table 2**)** and LIMS software (see Table 3) is device, whilst for SARS-CoV-2 serology measurements (see Table 4), it is either precoordinated pair or measurement, dependent upon the specific concept being described. The ‘standard_concept’ is null (non-standard) for all laboratory analyser systems, LIMS and SARS-CoV-2 serology measurements concepts, as the CO-CONNECT vocabulary is non-standard. ^68,74^

As part of the process, the CO-CONNECT concepts were, where possible, mapped to the representative standard SNOMED codes. These concepts can now be used to represent the respective devices and measurements within the OMOP CDM. Each concept has a unique ‘concept_code’ (source code) for each of the three CO-CONNECT vocabulary sections.^53^ These source identifiers follow the nomenclature of:

• CC-LAB-xxx for laboratory analyser systems;
• CC-LIMS-xxx for LIMS software;
• CC-SEROLOGY-xxx for SARS-CoV-2 serology measurements.

The CC stands for CO-CONNECT, whilst the xxx signifies an assigned integer, for example, 001. The fields of ‘valid_start_date’, ‘valid_end_date’ and ‘invalid_reason’ signify the lifecycle of a vocabulary. The ‘valid_start_date’ for all concepts was set as the first of December 2022 and the ‘valid_end_date’ was set as the thirty first of December 2099. There were no values for ‘invalid_reason’ as the vocabulary is currently valid. These concepts form part of the larger CO-CONNECT formalised vocabulary, which augments the currently available international standardised vocabularies, such as SNOMED.^68^

As data completion rates were low, it was necessary to specifically ask for the additional data points and national laboratories had to invest time and effort into providing these as it was not normal practice to share more than the result. This points to fundamental differences in the data requirements of the clinical and research use cases. Most NHS labs are configured to support clinical use cases and the additional data items required to provide research-quality data mean additional configuration, testing and work that cannot be justified within the clinical context. The paucity of implementable standards in this domain only makes it harder. Data points such as device type/id and the specific methodology used are not seen as critical/high value items in the clinical domain.

### Feasibility of adoption of the CSMDS

During the laboratory feasibility studies at Barts, GOSH and Tayside, it was found that it would be straight forward to change their laboratory systems to report the CSMDS granular data. Ten of the major LIMS software providers were contacted, introduced to the proposed CSMDS and asked to complete a survey on whether their software could accommodate the reporting of the CSMDS data attributes. Three LIMS software providers replied to the survey, with two stating that there would be no foreseen problems in implementing such a CSMDS, they could capture and report all of the data attributes and in fact were currently doing so for most assays. The third responded that instances of their software where setup differently with potentially different interfaces and therefore they were unsure if they could support the CSMDS. Further analysis is needed to fully understand at a national level what the barriers to implementation might be.

## Discussion and conclusions

The CO-CONNECT project investigated how COVID-19 serology laboratory results are captured and reported within the UK, focussing on how to improve these processes and how to capture more granular data. High-level qualitative results (positive, negative, etc.) were found to be routinely captured, yet many testing laboratories produce an array of quantitative data which could also be relatively easily reported. How data is reported via national systems such as Labgnostic (in England) and Scottish Care Information Store (equivalent system in Scotland) varies across different laboratories, ranging from full reporting to almost no reporting of quantitative data. This is in part due to how individual laboratories are configured, the analyser machines they utilise and the LIMS software they employ to capture, structure and then report said data. There was no nationally specified minimum set of data attributes for the reporting of COVID-19 serology results that laboratories could apply and adhere to, to support the reporting of granular results across the United Kingdom. The COVID-19 Serology Minimum Data Standard (CSMDS) fills this gap and sets out a clear and structured approach to what should be captured and reported, stipulating 12 distinct data attributes. The approach and method applied could be further refined and applied on a larger scale.

We found locally encoded naming conventions are often applied to data attributes, for example, the name given to the laboratory analyser system conducting the test. Utilising controlled vocabularies such as LOINC and SNOMED-CT resolves this variance supporting comparisons at a national level across assays, test kits, laboratory analyser systems and LIMS software. Our work has produced a set of formalised concepts as part of the CO-CONNECT standardised vocabulary for the representation of laboratory analyser systems, LIMS software names and SARS-CoV-2 serology measurements to support such an approach, with the view to understanding and potentially reducing variability of reporting testing data. These new concepts form the first step towards a standardised representation of laboratory analyser systems and LIMS software, not only for serology, but potentially for numerous other laboratory tests. Such an approach, if adopted nationally, can enable clear, unambiguous identification and reporting of the laboratory analyser systems, the LIMS software being used and the results for testing at a given point in time.

Historically, the granular level of detail has not been routinely captured as the calibrations and research questions generally have already been undertaken over many years prior to use in clinical care. COVID-19 was a new disease, and a rapid testing program was delivered within a clinical setting without prior research and calibration. To be able to respond in a timelier fashion to future pandemics and to support research requiring national level laboratory data linked to other relevant healthcare records, we highly recommend that standardised, granular level, laboratory data is captured nationally and shared using automated pipelines with organisations collecting other national health datasets. Although in England some high-level data is captured from several health trusts, granular level data is not fully captured and none of the information is currently shared and linked to other national health datasets via automated pipelines. Within Scotland, data is captured via the Sci-Store system but is also not standardised and automatically linked. When we investigated if granular level data could be captured at a Scottish national level, we were informed that the systems for capturing the data were antiquated and cannot be modified to capture more fields without risking stability.

Looking forward, there are three potential areas to address for this work to progress:

1. The work set out herein has utilised three NHS laboratories to gain a snapshot of the current situation, together with exemplar data from other CO-CONNECT partner NHS laboratories, thus it is not wholly representative of all organisations across the United Kingdom. This is therefore a limitation. Across England, there are multiple laboratories, using many different types of analysers and LIMS software, ranging from brand new state-of-the art systems to antiquated legacy systems. To facilitate adoption of the CDMDS, a thorough assessment of a wider range of organisations, technologies, software and reported laboratory messages must be performed to appreciate who does what, with what and how.
2. There is a need to apply a standardised vocabulary for the reporting of laboratory results. The use of more detailed standardised naming conventions (for laboratory analyser systems, LIMS software and SARS-CoV-2 serology measurements) will enable the reporting of higher quality detailed data. This will potentially allow for greater understanding of variability in laboratories which could support further work to address and decrease this.
3. To support future pandemics and research using test data, a new data pipeline should be built and configured, so that results can be reported to organisations such as NHS England.
